# Experiences implementing scalable, containerized, cloud-based NLP for extracting biobank participant phenotypes at scale

**DOI:** 10.1093/jamiaopen/ooaa016

**Published:** 2020-05-22

**Authors:** Timothy A Miller, Paul Avillach, Kenneth D Mandl

**Affiliations:** o1 Computational Health Informatics Program, Boston Children’s Hospital, Boston, Massachusetts, USA; o2 Department of Pediatrics, Harvard Medical School, Boston, Massachusetts, USA; o3 Department of Biomedical Informatics, Harvard Medical School, Boston, Massachusetts, USA

**Keywords:** natural language processing, medical informatics, phenotyping, biobanking

## Abstract

**Objective:**

To develop scalable natural language processing (NLP) infrastructure for processing the free text in electronic health records (EHRs).

**Materials and Methods:**

We extend the open-source Apache cTAKES NLP software with several standard technologies for scalability. We remove processing bottlenecks by monitoring component queue size. We process EHR free text for patients in the PrecisionLink Biobank at Boston Children’s Hospital. The extracted concepts are made searchable via a web-based portal.

**Results:**

We processed over 1.2 million notes for over 8000 patients, extracting 154 million concepts. Our largest tested configuration processes over 1 million notes per day.

**Discussion:**

The unique information represented by extracted NLP concepts has great potential to provide a more complete picture of patient status.

**Conclusion:**

NLP large EHR document collections can be done efficiently, in service of high throughput phenotyping.

## LAY SUMMARY

The text entered by physicians into electronic health records contains detailed information about patients, but it is more difficult to use in research applications than structured fields. Natural language processing (NLP) techniques can be used to convert text into more usable formats, but existing NLP tools do not scale to large collections, can be difficult to use, and existing solutions tend to be specific to single use cases or settings.

In this work, we developed an architecture to address all of these problems, using cloud computers (Amazon Web Services Elastic Compute Cloud) to address scaling, using containerization (Docker) to hide the complexity of NLP tools, and using open-source and standard tools (Apache cTAKES, Apache UIMA, Apache ActiveMQ) for a solution that should be widely usable.

We use this architecture to process the notes for all patients in a BioBank at Boston Children’s Hospital, with extracted NLP variables going into a database that can be queried by researchers. We then conduct experiments on a controlled subset of that data, and show that, for the range of scaling we explored, processing time scales nearly linearly with the number of cloud computers used to do the processing.

## BACKGROUND AND SIGNIFICANCE

Much of the potentially valuable information in electronic health records (EHRs) is “locked up” in unstructured text. NLP techniques can map the text into more usable formats, for example, SNOMED concept codes. NLP techniques for clinical data have substantially advanced in the last decade, but most NLP success stories involve one-off projects requiring substantial NLP and clinical expertise. A pipeline for large-scale processing of clinical text, with output made available to all clinical investigators at an institution, could increase the value of the EHR for research.

We sought to use an open-source NLP system, Apache cTAKES,^1^ to process the notes for a large biobank patient cohort at a pediatric hospital, as a first step in developing hardened processes and tools for processing the notes for the entire patient population. The goal of this processing is to make NLP phenotyping variables available to all researchers.

Other recent work has reported using cTAKES for processing large collections of notes. The first used similar components to this work—dictionary lookup to extract concepts and negation, applied to millions of notes.^2^ Another work used Apache Beam and Spark and cloud infrastructure to distribute the computation, again with standard components, applied to over 3 million notes.^3^ Another related effort wrapped MetaMap with database persistence, and Representational State Transfer (REST) web services as the interface to both processing and querying.^4^ The work we describe here differs in using containerization to encapsulate NLP components, extensive use of standard, and open-source technologies, and in that our code is made available on Github.

## MATERIALS AND METHODS

The desiderata that influenced our design were encapsulation, repeatability, and scalability. By encapsulation we mean hiding as many NLP details as possible from system users. To do this, we develop several standard NLP components in a container architecture called Docker, providing recipes for building these containers that eliminate the requirement to install and manage the cTAKES NLP software. At the same time, advanced users can tailor the scripts to create their own containers, requiring minimal effort for those who are already familiar with cTAKES. Repeatability is important because NLP research moves quickly, and we want it to be as easy as possible to run NLP pipelines with new components. Scalability is important because EHR systems can contain millions of records, and if we want to be able to repeatedly run different NLP systems, we aim to have their runtime be measured in hours and not weeks. Our scalability solution uses multiple technologies detailed below to allow users to simply specify how many CPU instances they would like to have running NLP pipelines.

The cTAKES software contains a number of modules that developers (including author T.A.M.) have created for different NLP tasks. These include linguistic pre-processing modules for breaking the text into sentences and tokens, and tagging the tokens with parts of speech (eg, noun, adjective). Next, candidate phrases are matched to a dictionary of concepts, by default SNOMED CT and RxNORM, and tagged with concept codes from the original dictionary, and a Concept Unique Identifier (CUI) from the NLM’s Universal Medical Language System (UMLS) Metathesaurus. This dictionary lookup is what many researchers want—putting text into a coded form that can be queried as easily as billing codes (eg, International Classification of Diseases, or ICD codes). The assertion status module then labels extracted concepts for whether they are *negated*, *uncertain*, *historical*, *conditional*, or *non-patient-related* (eg, family history).^5^

For this work, we developed a package of tools that extend the functionality of Apache cTAKES to easily scale on commodity cloud computing infrastructure. These extensions involve the use of three important technologies: (1) Apache UIMA-AS, an extension to UIMA to allow for distributed text processing, (2) Docker, a lightweight virtualization software, and (3) Amazon Web Services (AWS), and particularly the Elastic Compute Cloud (ec2), which allows for programmatically starting and stopping computing resources on demand. The combination of UIMA-AS and ec2 creates the scalability—different processing pipelines can be distributed between nearly unlimited numbers of servers. UIMA-AS can also be configured to allow for on-instance parallelization—we configured each ec2 instance to run two pipelines in parallel. Individual components may or may not take advantage of threading—we considered that outside the scope of this work, but our assumption is that most are single-threaded. Finally, Docker gives us encapsulation, as the UIMA-AS wrappers around cTAKES packages can be packaged as portable containers that can run on any hardware that runs Docker.

### Implementation

**Figure 1.**
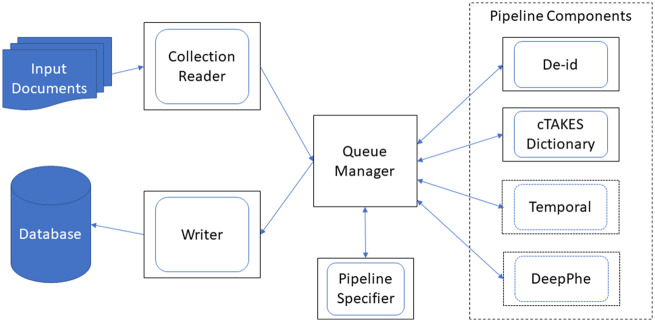
Architecture of scalable processing infrastructure. Solid blue represents storage devices. Outer black boxes represent Docker containers, inner blue boxes represent UIMA-AS components. Dotted lines (around Temporal^6^ and DeepPhe^7^) show potential future components, which we include to indicate where they fit conceptually in this architecture.

Figure 1 shows a schematic of the system architecture. The system architecture was split into a number of natural components, each of which is wrapped in a Docker container with a startup script. UIMA-AS provides tools that allow any component to be replicated multiple times for scalability, and we use ec2 instances provide the hardware for each replicated component. Specifically, the ec2 instances we use in this work are m5.large instances, which have 2 CPUs and 8 GB of RAM. The components we used include the following:

#### Document router

This container encapsulates the Apache ActiveMQ queue manager software. The cTAKES containers register with this component when they start up, so that when we pass a document to this component it will be routed through the correct components of the NLP pipeline.

#### Reader

This container reads from a given data source and passes the documents to the Document Router. We have implemented several example Readers that read from files in a directory, rows in an Oracle database, or documents in an Apache Lucene index.

#### NLP component(s)

This container type wraps an Apache cTAKES module that analyzes input text and adds some annotations to the data structure describing the document. Each annotator is wrapped in a UIMA-AS configuration file that specifies a network address of the cTAKES resource. A given annotator does not need to know where it belongs in a larger pipeline. We have implemented pipeline components for a de-identification module and the cTAKES dictionary lookup (which also includes negation processing). The de-identification module wraps the open-source MIST tool,^8^ using a statistical model trained on data from the SHARPn project.^9^ The dictionary lookup uses the cTAKES default, and we use the rule-based negation system that implements the ConText algorithm.^10^

#### Writer

This container type is typically the end stage for a pipeline. We have implemented writer containers for Oracle database in the i2b2/tranSMART format and for MongoDB in an ad hoc format, both of which are configurable with database credentials.

#### Pipeline specifier

This container type describes how to compose pipeline components into a complete NLP pipeline. It may also optionally include a writer component. We have implemented a pipeline that combines the de-identification component, dictionary lookup component (including concept negation classification), and writers to both Oracle and MongoDB databases.

### Evaluation

The architecture we described above was tested on a cohort of patients enrolled in the Boston Children’s Hospital PrecisionLink Biobank.^11^ These patients were broadly consented to allow biosamples collected during the course of treatment to be deposited into storage for future research, not yet specified. Phenotype is derived from EHR data collected as a byproduct of care. Patients with relevant phenotypes or genotypes can be discovered via the Portal query tool, which relies on the PIC-SURE API^12^ to interrogate a PIC-SURE High Performance Data Store (HPDS).^13^ Queries can be variant-first, phenotype first, or a combination.^13^ To complement the structured EHR data, we performed high throughput processing on clinical notes for 8239 patients (as of February 1, 2019), so that phenotype queries could include SNOMED concepts.

We ran our scalable version of cTAKES on AWS over the course of several days to monitor its progress and minimize any restarts due to unforeseen computational issues. We used a collection reader container that read from the Biobank’s central i2b2 database and a writer that wrote back to a different table in the same i2b2 database. This run focused on stability and monitoring (as opposed to scalability), using two nodes of the cTAKES dictionary lookup pipeline container and one node for every other container type. Following this run, we extracted a subset of 10 000 notes to an AWS filesystem, to run controlled experiments that would not subject the system to variation in performance if other users simultaneously accessed the i2b2 database. These notes had an average length of 851 word tokens, with the largest document having 21 195 tokens. We also performed analysis of the extracted CUIs from this experiment, including unique CUI counts, and statistics describing the distribution of semantic types of extracted CUIs (https://documentation.uts.nlm.nih.gov/rest/home.html).

In preliminary testing we found that our speed improvements were not linear with increases in scaling with ec2. To debug our scaling issue, we made use of a REST-based monitoring interface to the ActiveMQ queue manager package. This allows us to query the document router periodically for reports of queuing behavior. By monitoring these queues, we saw that the de-identification component consistently had larger queues, and so we added additional ec2 instances for that component.

## RESULTS

**Figure 2.**
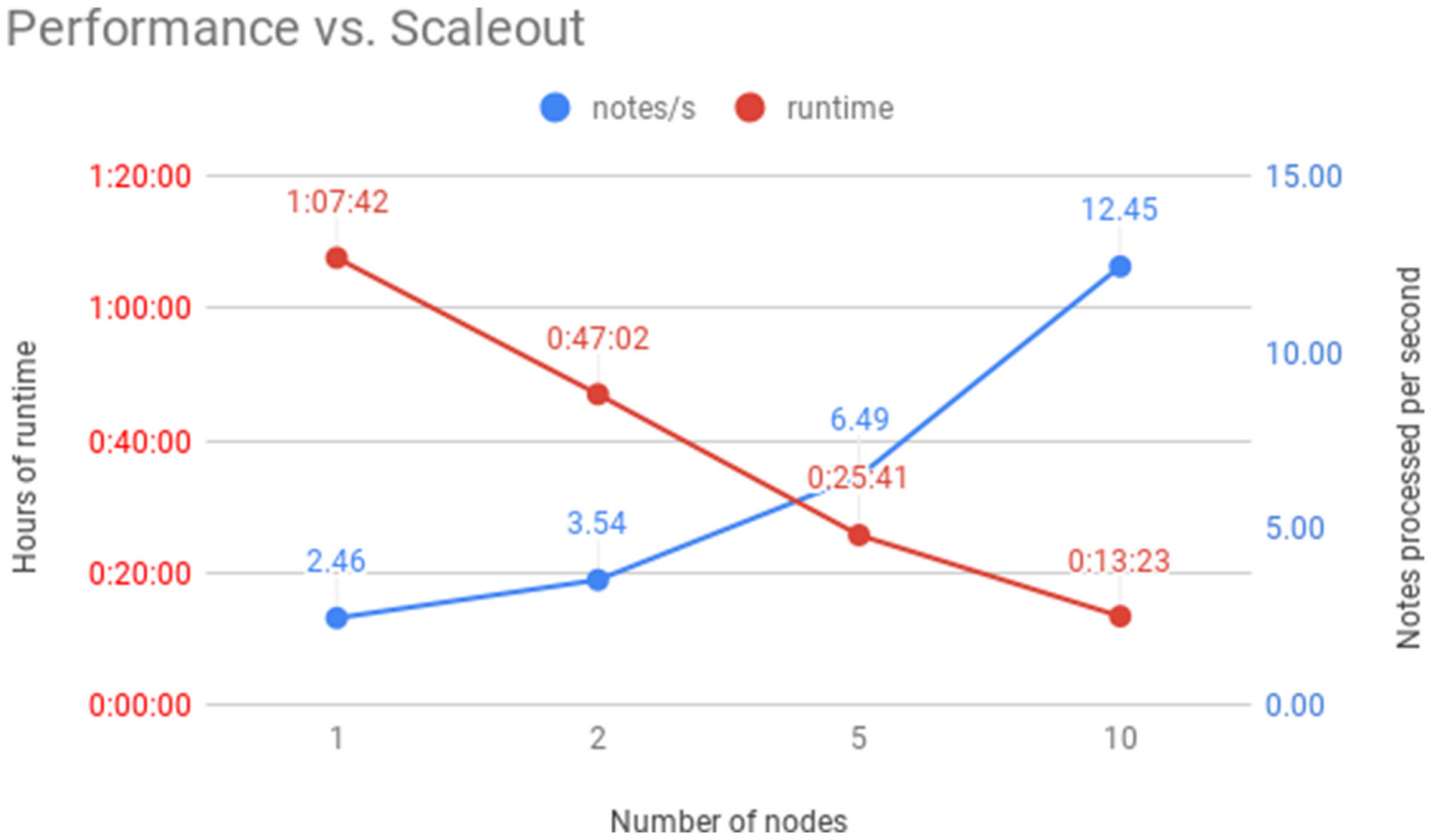
Performance plot, comparing number of m5.large computing nodes against processing time (left vertical axis) and processing speed (right vertical axis).

Figure 2 shows the results of different scale-outs for processing the 10 000 notes in a controlled scenario. We show a nearly linear speedup when going from one to 10 instances of each component. The fastest configuration is processing 12.45 notes per second. This corresponds to a speed of over 1 million notes per day, with the caveat that different input reading and output writing regimes may introduce new bottlenecks. The cost per million notes ranged between $31 for our cheapest setting, to $41 for the fastest setting.

### Outputs of processing

We processed more than 1.2 million notes associated with patients enrolled in the PrecisionLink Biobank, finding over 154 million concepts. These were written to an Oracle database, which were then incorporated into the user interface, so that codes extracted from NLP could be used analogously with structured information (ICD codes) in cohort exploration tools.

**Figure 3.**
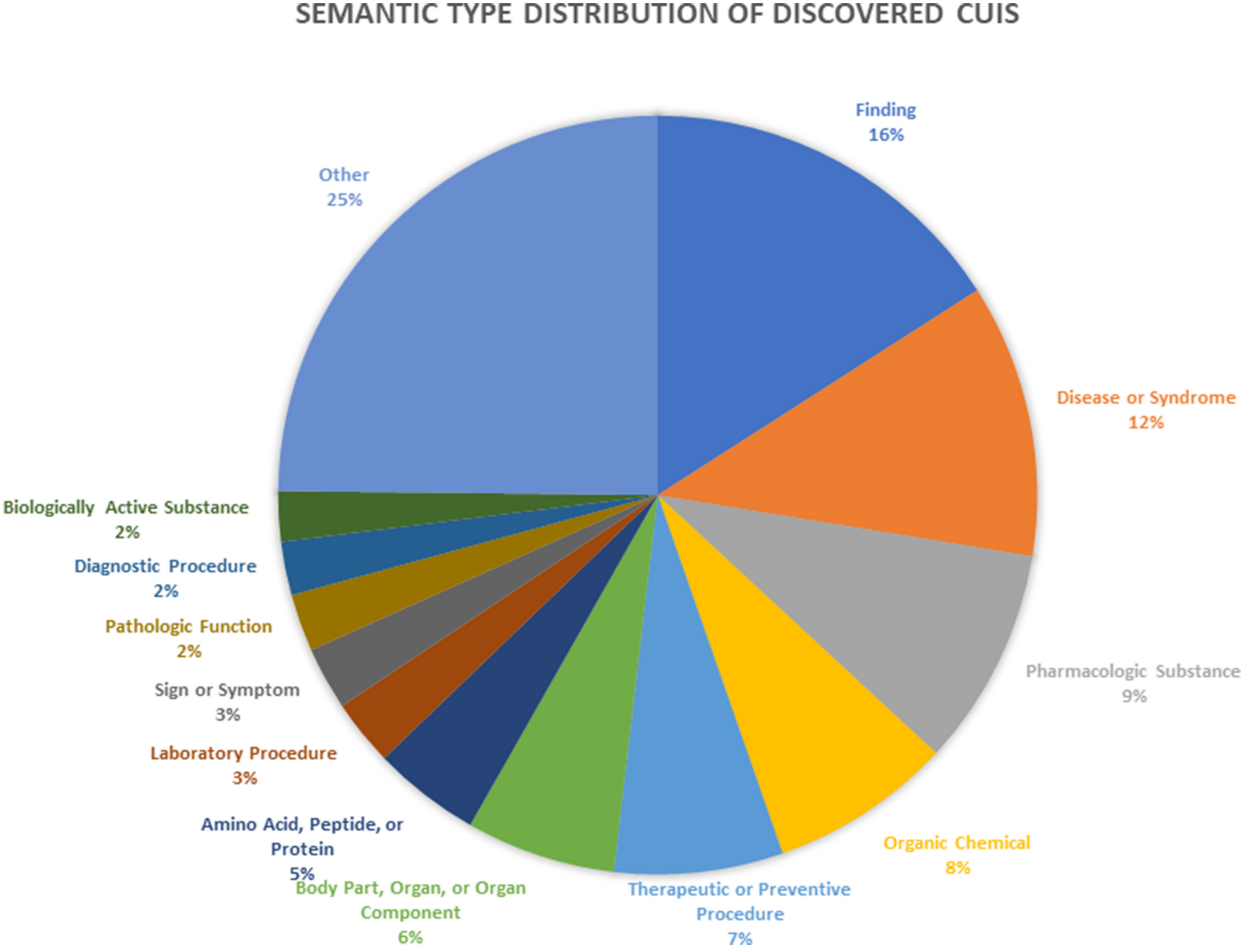
Pie chart showing proportions of different UMLS semantic types represented by extracted CUIs.

In our more controlled experiment that processed 10 000 notes, the system discovered 14 998 unique CUIs (comprising 394 million CUI instances). Figure 3 shows the proportions of different semantic types represented by the extracted CUIs.

## DISCUSSION

We were able to engineer our framework so that in our controlled experiment, scaling occurs nearly linearly with the number of ec2 nodes used. One of the most important practical considerations is scaling even further to allow for processing of massive collections. Boston Children’s Hospital has more than 70 million notes in total that could be processed, and at the fastest speed we report here it would take 65 days to process them, at cost of $2870 using the fastest $41 per million notes estimate from above. Our reported results suggest that to process all of the BCH notes in a week with this configuration, it would require a scaleup to 93 ec2 nodes. Diminishing returns of scaleup are likely, however, as various factors make purely linear scaling difficult to sustain, so this should be seen as a lower estimate. Experimenting with various ec2 instances and other parameters could potentially change these figures; deeply exploring the tradeoffs between processing time and costs will be important for repeated institution-scale processing.

Our results also show a potential significant potential value in NLP. Important semantic types like Finding, Body Part, and Sign or Symptom make up many of these concepts, and these types provide important information regarding patient status that may not be represented anywhere in codified data. It is difficult to rigorously quantify the added value of NLP over codified data, since every institution may use different coding systems, and capture different levels of granularity. However, in the months following the processing described here, we worked with many interested investigators and report three example queries that NLP permitted that codified data did not: ketotic hypoglycaemia, for which the dictionary lookup identified the SNOMED code, and the negation detector found rule-outs; opsoclonus myoclonus syndrome, a rare disease without an ICD9 code that was identified by the NLP dictionary lookup; and focal epilepsy, for which our coding was not specific enough to capture, but which again the dictionary lookup on the text was able to capture and map to SNOMED.

This is especially valuable in identifying patients with rare diseases, where ICD may not have a code for the disease, but where NLP captures the disease either through a SNOMED mapping or through some combination of findings and signs and symptoms.

## CONCLUSIONS

This work shows that it is possible to build scalable, containerized, cloud infrastructure for processing clinical notes in an EHR system. The resulting infrastructure is more suitable for repeated runs, for example when new NLP components are developed. The modular nature of the architecture allows implementers to mix and match readers and writers for custom setups. The code developed for this work is available under the Apache 2 license at https://github.com/tmills/ctakes-docker.

### FUNDING

This work was supported by U01TR002623 from the National Center for Advancing Translational Sciences, NIH, by U01HL121518 from the National Heart Lung and Blood Institute, NIH, by R01LM012973 from the National Library of Medicine, and by the Boston Children’s Hospital PrecisionLink Biobank.

### AUTHOR CONTRIBUTIONS

T.A.M. implemented the methods, ran the experiments, and drafted the manuscript. T.A.M., P.A., and K.D.M. designed the architecture and interfaces, and edited and revised the manuscript.

## CONFLICT OF INTEREST STATEMENT

None declared.
